# Implementing a smoking cessation service in routine and manual workplaces: A qualitative service evaluation

**DOI:** 10.1016/j.puhip.2026.100862

**Published:** 2026-10-03

**Authors:** Amelia Talbot, Nicola Lindson, Jonathan Taylor, Angela Difeng Wu

**Affiliations:** Nuffield Department of Primary Care Health Sciences, University of Oxford, Radcliffe Primary Care Building, Radcliffe Observatory Quarter, Woodstock Road, Oxford, OX2 6GG, UK

**Keywords:** Smoking, Smoking cessation, Workplace, Health inequalities, Qualitative evaluation

## Abstract

**Objectives:**

Routine and manual workers have higher smoking rates than those in professional occupations, contributing to health inequalities. Workplace cessation interventions may increase long-term abstinence in this group, but employee uptake is low, and managers often oppose onsite delivery. We aimed to understand the barriers and facilitators to implementation and employee uptake of a workplace smoking cessation service from the perspective of cessation advisors, workplace managers, and service coordinators. The service was a free 12-16-week programme of weekly behavioural support plus nicotine replacement therapy, medication, or an e-cigarette.

**Study design:**

Qualitative service evaluation.

**Methods:**

One cessation advisor, one workplace manager, and two service coordinators (one from the council and one from the service provider) participated in semi-structured interviews. Data were descriptively analysed against the four constructs of Normalisation Process Theory.

**Results:**

Coordinators reported misunderstandings about the scope of the service and which workplaces were eligible, alongside employer concerns about perceived administrative and financial burdens (Coherence). Concise communication and council involvement helped navigate organisational gatekeepers, although outreach was constrained by limited dedicated capacity (Cognitive Participation). Workplace factors, including lack of private space and shift-work patterns, reduced employee uptake (Collective Action). Participants also highlighted threats to long-term sustainability, including limited anonymised feedback to managers on uptake and cessation outcomes and the need for repeated managerial approvals for onsite delivery (Reflexive Monitoring).

**Conclusions:**

Implementation of smoking cessation services within routine and manual workplaces may hinge on improving communication between those who commission and deliver services and the workplaces they seek to support.

**What this study adds**

• Service coordinators and a worksite manager perceived that implementation of a workplace smoking cessation intervention was driven by the intervention being free and potentially being beneficial to businesses and employees.
• However, they believed that implementation was limited by miscommunication on service scope and what classifies as a routine and manual workplace.
• Coordinators also believed that employee uptake may be hindered by shiftwork and a lack of confidential space.

**Implications for policy and practice**

• Our service evaluation suggests that there is a need for clearer communication between those who commission and deliver workplace smoking cessation services.
• Our evaluation also highlights that smoking cessation services may need to be tailored to the varied contexts of routine and manual work.

## 1 Introduction

Cigarette smoking has consistently declined in the UK, yet rates remain disproportionately high among routine and manual workers. [1] These occupations, including construction and manufacturing, often involve physical labour, repetitive tasks, and require varying levels of educational/vocational training. [2]

The 2021 Census found that 28.9% of people aged 16+ were routine and manual workers, [3] with 18.8% smoking compared to 6.5% of people in professional roles, contributing to smoking-related health inequalities. Routine and manual workplaces can have a culture where smoking is a social and accessible activity, especially during outdoor work, and a coping mechanism for stress and boredom. [4]

While evidence-based cessation interventions are offered in general practice, routine and manual workers, particularly men, may be less likely to access them. [5] Workplace interventions may help overcome barriers associated with attending healthcare services by embedding support within environments that workers already access regularly. Systematic review evidence shows that interventions incorporating a range of components, including mindfulness, behavioral counselling, health education, and interventions by social media, have a positive effect on smoking cessation. However, employee uptake is often low and navigating organisational gatekeepers, including managers, to agree to implement these services in their workplaces can be a key challenge. [6]

Understanding the barriers and facilitators to implementing workplace smoking cessation services—including how to secure buy-in from organisational gatekeepers—is increasingly important in the context of UK tobacco control policy. We were commissioned by a council in England to conduct a service evaluation of a workplace smoking cessation service for routine and manual workers. This initiative was in response to the UK Government's *Smokefree Generation* policy, which encourages local authorities to increase engagement with groups who may be less likely to access cessation services. [7]

We conducted a qualitative service evaluation to understand the barriers and facilitators to implementation and employee uptake of this service from the perspective of workplace managers, cessation advisors, and service coordinators.

## 2 Methods

This study was designed as a qualitative service evaluation of a workplace smoking cessation service implemented by a local council in England. Our methodological positioning was qualitative descriptive, as we aimed to identify and learn from participants' experiences to provide actionable recommendations to the council on improving implementation, rather than to generate theory.

### 2.1 The service

The council commissioned a private healthcare provider to deliver an onsite workplace smoking cessation service for routine and manual workers. The service offered a free 12–16-week programme combining weekly behavioural support from trained cessation advisors with a choice of one quitting aid (e-cigarette, nicotine replacement therapy, or licensed medication). By February 2026, the service was integrated into seven sites: one health care service and six customer service providers (including supermarkets).

AT conducted a one-day visit to a customer service provider in September 2025 to observe an advisor delivering the intervention in real-time. Decisions by workplaces to integrate the service were completely separate from their choice to participate in the evaluation.

### 2.2 The evaluation

We were commissioned to evaluate the effectiveness and acceptability of the workplace cessation service between September 2025 and February 2026. Our protocol proposed using mixed-methods to examine 14 outcomes, including employee uptake, quit rates, and employee experiences with the service (measured qualitatively with semi-structured interviews) [Supplementary Material 1].

The service provider was responsible for obtaining consent from workplaces to participate in the evaluation. However, none of the workplaces taking part in the scheme agreed to participate, meaning we could not recruit any employees to our evaluation. Reasons reported by the provider pertained to communication barriers, including workplaces not responding to email correspondence and difficulty establishing contact with senior management to consent to the evaluation at larger workplaces. As these challenges were themselves relevant to understanding implementation, the evaluation was pragmatically refocused toward exploring barriers and facilitators to workplace engagement and service delivery. Qualitative interviews were therefore conducted with service coordinators involved in workplace recruitment, alongside any cessation advisors and workplace managers.

### 2.3 Sampling and recruitment

Participants were recruited opportunistically. The provider invited managers and advisors via email, while AT directly emailed the coordinators from the council and service provider. Those that showed interest were emailed by the research team with an information sheet outlining what participation involved, the ethics waiver, potential risks and benefits, their right to withdraw up until a certain time point, and how we would use their data (including the possibility that anonymised excerpts from their interview might be published in scientific journals).

As the authors had a prior professional relationship with the coordinators, there was an increased risk of social desirability, wherein participants respond with what they perceive the interviewer wants to hear. To manage this, the interviewer asked all questions on the topic guide, avoided assuming prior knowledge of participants' answers, used open-ended questions, and reminded participants that there were no right or wrong answers and that their honesty was important to the evaluation.

### 2.4 Data collection

Participants were invited to one-to-one, remote, semi-structured interviews. Topic guides were developed for each participant group by AT (female sociologist) and reviewed by ADW and NL (female smoking cessation experts), though not piloted [Supplementary Material 2].

Interviews were conducted by AT in February 2026, lasted 1 h, and were audio-recorded and transcribed verbatim. Verbal consent to participate was taken at the start of the interview and before starting the audio-recording. Participants did not review transcripts and field notes were not taken to allow AT to focus on the participants’ responses. The researcher stopped interviewing when all recruitment avenues had been exhausted and the evaluation period ended. This represents a pragmatic stopping criterion limited by the time constraints and resources available to the research team [8], which was appropriate in this evaluation given the low uptake of interviews.

### 2.5 Data analysis

In March 2026, AT conducted a rapid analysis to identify implementation challenges at the semantic level and provide and receive formative feedback on our findings from the council. In May 2026, the researcher performed a thematic analysis to capture descriptive patterns across the dataset. Using the manual ‘One-Sheet of Paper’ method, [9] AT mapped the data and identified that the findings aligned with Normalisation Process Theory (NPT) [10] [Table 1]. AT mapped the data against each construct and made a descriptive theme for each. To enhance trustworthiness, AT engaged in reflexivity throughout, critically evaluating how her subjectivity, values, and prior professional relationship with some participants may have shaped data interpretation. Ongoing findings and interpretations were discussed through iterative peer coding clinics with ADW and NL. Differing perspectives on the data and implications for practice were discussed until agreement was reached, and all co-authors reviewed the final manuscript.

**Table 1:** The constructs of normalisation process theory.

Normalisation Process Theory is a sociological and implementation framework used to explain and evaluate the ‘work’ that people do to ‘normalise’ (routinise) new interventions into existing organisational practice. This work is represented by four constructs that can act as barriers or facilitators to implementation.		
Construct	Definition	Question Asked of Our Data^a^
Coherence	The sense-making work to understand the purpose and scope of the service.	Do participants have a shared understanding of the service?
Cognitive Participation	The relational work to navigate organisational gatekeeping.	What was the process of facilitating workplaces to ‘buy-into’ the service?
Collective Action	The operational work to enact the service into the workplace.	What workplace logistics supported or prevented the delivery of the service?
Reflexive Monitoring	The appraisal work to assess the long-term impact and sustainability of the service.	How did participants evaluate the success of the service and its potential for long-term implementation?

a Questions were adapted from Murray E, Treweek S, Pope C, MacFarlane A, Ballini L, Dowrick C, Finch T, Kennedy A, Mair F, O'Donnell C, Ong BN. Normalisation process theory: a framework for developing, evaluating and implementing complex interventions. BMC Medicine. 2010; 8(1):63. DOI: https://doi.org/10.1186/1741-7015-8-63.

## 3 Results

One smoking cessation advisor who worked across multiple customer-service locations, one workplace manager from a health service provider, and two service coordinators (one from the council and one from the service provider) were interviewed (n = 4). The resulting four themes are presented below. A coding tree is in Supplementary Materials 3.

### 3.1 Coherence: misunderstandings of the service

A barrier to implementation was a lack of coherence between advisors and coordinators on the service scope. While intended for employees, our site visit and reports from the council highlighted that the service was delivered to customers as well as—or instead of—employees. This was also evident during our interview with the cessation advisor. For example, the advisor suggested that client retention was improved through flexible scheduling and in-person consultations. He perceived that in-person meetings motivated clients to quit by offering a visual marker of progress through carbon monoxide monitoring. However, these strategies were discussed in the context of customers accessing the service within routine and manual settings instead of solely worksite employees.

This disconnect extended to what qualified as a ‘routine and manual’ workplace; for example, the provider included a certain customer service organisation under the commission, whereas the council did not recognise this as an eligible workplace. The participant highlighted that the service was delivered within an area of the workplace accessible to the general public, which may have included routine and manual workers, rather than being targeted specifically at employees:

“*I don’t really want to count that* [customer-facing organisation] *as a workplace because I think it’s just the prime spot for routine and manual workers and just the general population … I’ve challenged them* [the provider] *on that.” (Council Coordinator)*

Challenges with coherence also presented during site recruitment. Both coordinators recalled that some workplaces declined the service due to assumptions that it would impose an administrative burden, or financial costs to the business and employees and that this often-needed clarification:

“*What I come across a lot is that people don’t know it’s a free service … There are quite a few* [who] *are quite shocked that actually, no, if you’re in the service and you’re engaging, you don’t have to worry about paying for anything because it’s all covered.” (Provider Coordinator)*

### 3.2 Cognitive Participation: navigating organisational gatekeeping

The relational work involved in engaging the ‘right’ manager at a low-stakes time was described as a major implementation challenge by both coordinators. This was particularly time-consuming in larger workplaces, where both coordinators described frequently chasing-up managers who initially expressed interest but failed to follow up or pass-on information. This challenge was exacerbated when plans to recruit a dedicated outreach officer for relationship-building with local businesses and site recruitment were unsuccessful, as reported by the council coordinator:

“*We were going to recruit a specialist tobacco dependency advisor that was going to focus on workplaces, but there were a lot of issues with that* [to do with short-term contract] … *I think not having someone that’s dedicated specifically for that made it difficult to recruit workplaces” (Council Coordinator)*

Both coordinators used several strategies to navigate this organisational gatekeeping. They kept their communication persistent and concise, involved the council to lend the service credibility and authority, and provided evidence of the potential benefits of workplace cessation interventions:

*“I believe when* [the service provider]*, when they sent them* [workplace] *an e-mail, they didn’t respond. But once I sent an e-mail* [from the council email]*, they did respond … I think it’s the authority bias, isn’t it? It’s the authority bias. They see gov.uk e-mail and then to them, they’re like, oh, I think I need to respond to this.” (Council Coordinator)*

### 3.3 Collective Action: managing workplace logistics

The delivery of the intervention was shaped by the context of each workplace. For example, the manager said that the service was successfully delivered within their workplace's confidential ‘staff wellbeing center’. Other customer-facing businesses, however, declined the service due to a lack of private space, as reported by the coordinators. The service coordinator explained that he occasionally negotiated this barrier by collaborating with managers to identify alternative spaces where the service could be delivered. These included using the service provider's tour bus in company car parks or operating within customer-facing areas (as observed in our site visit). However, both coordinators expressed concern that these alternative arrangements compromised privacy and might have hindered employee uptake. We also noted during our site visit that delivering the service in customer-facing areas seemed to attract only customers rather than employees.

These logistical barriers were compounded by the fact that the service was offered exclusively during routine working hours, which was incongruent with the schedules of shift workers, as reported only by the manager:

“*It would be more variety of appointments* [to improve the service] *… varying the days that those appointments happen would be really useful because of the nature of* [workplace] *shifts. Typically, an* [employee] *would work 37 hours, but they’d work over three 12 and a half hour shifts. They might work Monday, Tuesday, Wednesday, and then be off Thursday, Friday, Saturday, Sunday. If I only ever offer appointments on the Thursday, because that’s the only date the provider is available to give me the sessions, but they* [employee] *only ever work Monday, Tuesday, Wednesday* [so they cannot access the service].” *(Manager)*

Factors perceived as supporting employee uptake according to the manager and both coordinators included advertising the service extensively through existing channels.

### 3.4 Reflexive Monitoring: long-term sustainability of the service

When asked to appraise the potential for long-term sustainability of the service, the manager said that while it was “*a good service to have*” and “*something I want to maintain in some form*,” it was “*impossible to say*” if he would do so. This was because a lack of communication from the provider on staff uptake and cessation outcomes left him unable to assess if the service had a positive impact on employees. Comments unique to the advisor also highlighted that a lack of contingency between managers may threaten the long-term sustainability of the service:

“*We’ve got* [customer-facing organisation] *that’s been there from us doing it in the car park for quite some years, then we got in the café, talking to the duty managers to get the okay and there were no issues. Unfortunately, the community champion made it quite hard to be there … If we want to go back to* [customer-facing organisation], *we now need like a letter from head office.” (Cessation Advisor)*

## 4 Discussion

While systematic review evidence shows workplace smoking cessation interventions had a positive effect on abstinence, [11] our evaluation highlights that implementation hinges on organisational factors that compound service-user-level barriers like motivation and addiction. [12] Specifically, non-coherence between the intended and actual service scope may mean that employees already facing barriers to smoking cessation support in general practice will miss out on the opportunity to access it at their workplace. [5] This suggests a need for alignment and clear communication between those who commission and deliver smoking cessation services.

As elsewhere, [13] misunderstandings about the practical demands of the service appeared to influence workplace engagement. Participants observed employers assuming the service would involve financial costs or substantial administrative burden, with perceived interest increasing once coordinators clarified that the service was free. Our observations also align with research into the links between word choice and the uptake of health interventions, which found that using the word ‘free’ early in a discussion led to more efficient referrals. [14]

The heterogeneity of routine and manual workplaces may help explain why there was variation in the challenges described when enacting the intervention into workplaces. While small workplaces may lack a confidential space to host these interventions, previous work has supported the manager's observation that shift work and limited flexibility in break times also constrained uptake of the intervention. [6] This variation points to the potential value of tailoring interventions to specific workplace contexts rather than treating routine and manual workers as a homogeneous group.

### 4.1 Strengths and limitations

Our findings offer practical considerations for embedding smoking cessation services in routine and manual workplaces, but the small sample size (n = 4) lacked the information power to provide a comprehensive view of implementation barriers and facilitators. [15]. To our knowledge, this is the first study to use NPT [10] to organise data on the barriers and facilitators of implementing workplace smoking cessation interventions. We interviewed all coordinators responsible for recruiting sites, but findings may not reflect the views of all managers who agreed to the service or the cessation advisors delivering it. Participants’ perspectives represent their hypotheses on why employers declined/agreed to the service. We were also unable to learn directly from the employees who had access to the service, or understand what specific barriers were experienced by different types of employees and different workplaces settings and why.

We were unable to report sociodemographics to avoid participants becoming identifiable given the small sample size, which limits the transferability of our findings. Future research involving a larger sample could collect and report this data to assess how sociodemographic factors and specific workplace contexts might influence the implementation of smoking cessation interventions for routine and manual workers.

## 5 Conclusions

In conclusion, implementing smoking cessation interventions within routine and manual workplaces may be shaped by organisational alignment on service scope, workplace capacity to provide confidential delivery spaces, and sustained communication between commissioners, providers, and employers. Addressing these barriers, alongside tailoring interventions to the diverse contexts of routine and manual work, may help improve implementation and employee engagement with workplace smoking cessation support.

## Ethical approval

The project was classified as service evaluation by the Central University Research Ethics Committee at the University of Oxford, and formal ethics review was therefore not required.

## Data availability statement

The initial protocol for this service evaluation, interview materials, additional supporting quotes, and a mapping of the thematic analysis to the original rapid analysis are in the supplementary materials. Participants provided verbal consent for anonymised excerpts from their interview to be published in this manuscript. Full transcripts are not publicly available, as we do not have the participants’ consent for sharing their data in this way.

## Declaration of generative AI use

The authors wrote the initial draft of this manuscript in its entirety. Google Gemini was then used in parts of this manuscript solely to refine sentence structure and for conciseness to meet the journal's word limit. Following use of Gemini, the authors reviewed and edited the content and accept full responsibility for the content of the published article.

## Funding

This service evaluation was funded by a local council in England [grant recipient: NL]. At the request of the council, we have anonymised their identity. AT was funded by the National Institute for Health and Care Research (NIHR) Applied Research Collaboration [ARC]. The views expressed are those of the author(s) and not necessarily those of the local council, NIHR, or the Department of Health and Social Care.

## Declaration of competing interest

The authors declare that they have no known competing financial interests or personal relationships that could have appeared to influence the work reported in this paper.
