# Supplementary material for: Implementing a smoking cessation service in routine and manual workplaces: A qualitative service evaluation
Source: Public Health Pract (Oxf). 2026 Oct 3;12:100862. doi: 10.1016/j.puhip.2026.100862 (PMC13678017; doi:10.1016/j.puhip.2026.100862)
Supplement: Supplementary file 1 [file mmc1.docx]

## Supplementary File 1 – Service Evaluation Protocol

# A protocol to evaluate a local council’s workplace tobacco dependency services

Background

As part of the Tobacco and Vapes Bill^1^, local authorities have been encouraged to do more to (a) encourage people to set a smoking quit date with local stop smoking services and to (b) engage with people who smoke who would not ordinarily engage with stop smoking services.

A [redacted] private healthcare service provider is currently commissioned by a local council in England to support people to quit smoking. Their standard offer comprises three levels of support based on the components of the Robert West Smoking+ Model^2^ and are compliant with the National Centre for Smoking Cessation and Training (NSCST) Standard Treatment Programme^3^ and NICE guidelines^4^.

Level 1: provides digital-based information and support for quitting alone.

Level 2: provides brief advice plus pharmacotherapy.

Level 3: provides specialist stop smoking support, including up to 12 weeks of nicotine replacement therapy (NRT), cytisine or varenicline, or up to 9 weeks of bupropion.

Activities delivered through levels 2 and 3 are required to provide a minimum of 400 four-week quits per annum. All people wishing to quit are triaged so they can make an informed choice about the differences in the interventions provided at each level, the likelihood of a successful quit and the commitment required. Typically, people in the high-risk, priority groups, are encouraged to seek support at Level 3.

The service provider is not permitted to purchase unlicensed medications (such as e-cigarettes) from the pharmacotherapy budget; however, they are currently accessing free 4-week vaping starter kits via the government’s Swap to Stop Scheme^5^. This enables them to offer a voucher for a starter kit to any client, alongside alternative NRT products, cytisine, varenicline or bupropion as options to help them quit. The local council have produced a “Vaping Position Statement”. This statement, founded on the principle of informed choice, highlights the evidence on the effectiveness of nicotine-containing vapes in quitting smoking, while also acknowledging potential health risks that are not yet fully understood.

The service is accessible 7 days a week, 52 weeks a year (excl. public holidays) and during daytime, evenings and weekends.

## Plans to expand stop smoking services

The local council plan to use funds designated to improve stop smoking service engagement and increase quit rates to build the stop smoking service’ capacity and provide innovative services in locations most likely to reach smokers. The goal is to achieve approximately 332 additional ‘Set A Quit Dates’ in 2025-26, resulting in an additional 200 four-week quits (assuming 60% of those who set a quit date remain successfully quit at four-week follow-up). Therefore, the service provider will be awarded funding to employ three additional tobacco dependency advisors (TDAs), and additional services and very brief advice (VBA)+ ^6^ training will be implemented. This extra support will target three key groups who have higher smoking rates than the general population and/or are currently underserved by the existing service, as follows:

1. People living in the community with mental health conditions: currently people receiving in-patient care for mental health conditions who smoke are offered tobacco dependency treatment and provided with a vape on admission, but this support stops once they are discharged into the community. The new service will enable the service provider to work more closely with the community mental health team and other related NHS partners (such as the physical health nurses and the Health Outreach Liaison Team nurses) to support service users to remain abstinent from smoking or to continue trying to quit smoking while transitioning from secondary to community care. It is acknowledged that the service provider can take a more nuanced approach to vaping products with this cohort, where evidence from the Mental Health Trust suggests that people are likely to want to continue to vape straightaway, if it has helped them. The consultants in public health for the local council are in agreement with this nuanced approach for this cohort.
2. Postpartum women and their live-in partners/carers: currently pregnant women who smoke tobacco are signposted to a TDA embedded in the maternity unit. However, this support stops once the person has given birth and the partners of pregnant women are not offered tobacco dependency treatment. The planned new service will support postpartum women and their live-in partners who smoke up to 12 weeks postpartum.
3. People who work in jobs classified as routine and manual: the current service achieves around 40% of all its four-week quits in people classified as routine and manual. However, the prevalence of smoking in this cohort remains high in the local council catchment area [redacted statistics showing prevalence]^7^. The service is currently limited to providing leaflets and cards to relevant workplaces and tends to engage with this cohort outside of people’s actual places of work (such as, in clinics held in car parks at supermarkets, at [redacted] College and in other locations within areas of higher deprivation). The local council plan to enhance this offer by providing a tobacco cessation programme to staff within routine and manual workplaces and working with employers to develop smoke free policies, to promote and embed access to tobacco dependency support in the workplace and to promote to employers the benefits of having a smoke free workforce.

This protocol and the methods below relate specifically to an evaluation of the new services proposed in routine and manual workplaces (point 3 above).

## Programme development

In the first instance, the local council have partnered with the behavioural insights organisation [redacted]. They have conducted focus groups and semi-structured interviews with staff members within routine and manual workplaces to develop personas that capture the beliefs, behaviours, and motivations of individuals who smoke within this setting. This work has informed the development of the new tobacco dependency services and materials that will be targeted at routine and manual workers, who are disproportionately affected by tobacco dependency and may struggle to access conventional support due to irregular work patterns, workplace cultures, or limited awareness of available services. This new service will be implemented in June 2025 and provide designated tobacco dependency advisors who will provide the following:

- Workplace and Community-Based Outreach: advisors will deliver cessation support directly in workplace or community settings to remove access barriers.
- Flexible Appointment Options: appointments will be adapted to fit around non-standard working hours and shift patterns.
- Employer and Trade Union Engagement: advisors will work collaboratively with employers and trade unions to integrate smoking cessation into workplace wellbeing policies and cultures.
- Behaviourally Informed Communications: messaging and engagement strategies will be informed by local behavioural insights to ensure relevance and effectiveness for the target group.

## Evaluation methods

### Objectives

1. To evaluate the acceptability and feasibility of the new routine and manual worksite tobacco dependency programme being implemented by the local council and the service provider within routine and manual worksites.
2. To evaluate the level of engagement with the programme, the number of quit dates set and the number of four-week quits.

### Populations of interest

1. Routine and manual workers
2. Employers/management teams at the routine and manual worksites
3. The service provider TDAs delivering the service

### Outcomes

1. Employers’ baseline worksite smoking and vaping practices and policies (e.g., whether smoking status is recorded, whether smoking/vaping is allowed in certain areas of the site, policies on smoking breaks)
2. Employees’ opinions of their employer, regarding their approach to employees who smoke (including how this has changed from before implementation of the new approach to following the implementation)
3. Successful implementation of aspects of the programme
4. Acceptability (employers, employees, TDAs)
5. Uptake of support by employees
6. Number of quit dates set by employees
7. Quitting aids used by employees
8. Employees four-week quit rate (biochemically validated using CO)
9. Employees twelve-week quit rate (biochemically validated using CO)
10. Any changes (or intended changes) to worksite smoking policies (examples given in point 1) at evaluation end
11. Factors that influence the ability of employees/management teams to implement the service
12. Factors that influence the ability of TDAs to provide the service
13. Factors that influence the uptake of support to quit smoking employees
14. Factors perceived to be influencing the success of the service (employers, employees, TDAs)

### Evaluation methods

The evaluation of the tobacco dependency services provided by the service provider*,* in collaboration with the local council, will employ a mixed-methods approach, incorporating quantitative and qualitative evaluation methods to comprehensively assess the outcomes above and to add depth and breadth to participants’ experiences. All outcomes will be assessed after the service has been implemented; approximately 8 weeks or longer after initial implementation. This section outlines the specific methods that we will use to investigate these outcomes.

#### Recruitment

1. TDAs: The service provider or the local council will send contact emails of TDAs to [author one] (University of [redacted] researcher) over email. [author one] will then approach the TDA over email with an information form to look at regarding the potential to take part in an interview. If TDAs express an interest in being interviewed an interview will be arranged at a time of their convenience. Each TDA will have completed at least one advisory session before being interviewed.
2. Employers/ management team members: On recruitment of sites, the service provider or the local council will ask employers if they willing to be contacted by the University of [redacted] to take part in the evaluation. If they agree, the service provider or the local council will send contact details to [author one] over email. [author one] will then approach the employer or team member over email with an information form about the project and ask if they are happy to take part in an initial survey over the phone about their workplace and smoking policies. During the phone call they will be offered the opportunity to take part in a follow-up in-depth interview at a time of their convenience.
3. Service users: Recruited employers will share a link to a survey created by the University of [redacted] with their employees. As part of that survey employees will be provided information about a follow-up interview, which they can opt into by providing their contact details. [author one] will then approach the service user over email with an information form and arrange an interview at a time of their convenience.

Incentives such as prize draws or individual rewards will be offered to increase response rates, as preferred.

#### Quantitative

Outcomes 5-9 are routinely collected by the service provider. This data will be anonymised and shared with [redacted] as part of this evaluation.

All other outcomes will be assessed through a mixture of quantitative and qualitative measures in an attempt to capture as much data as possible from a range of people. Quantitative data will be collected via online surveys, from all of the populations. Surveys will be advertised using methods advised by the workplace, which could include leaflets, posters, email, paper copies on site. The surveys will include questions on smoking behaviour, service utilisation, satisfaction levels, barriers to access, and the perceived impact of the services. No identifiable data will be collected.

#### Qualitative

We will collect qualitative interview data, using a purposive sample of up to ~20 service users. We will also convenience sample ~5 TDAs to understand their experiences of delivering and using the service and ~5 employers/ management team members to understand how they found having the service embedded in the workplace and how they might encourage employees to use the service. However, following information power, we might stop interviewing sooner if the data collected is sufficient and high-quality enough to satisfy the evaluation’s aims and in line with practical circumstances^8^.

We will create a sampling frame for the purposive sample, identifying a variation of people that we want to talk to across ages, ethnicities, genders, job types, site, and other relevant smoking behaviour information collected by the survey measures. Following best practice, the frame will be iterated as needed in discussion with the evaluation team and as the evaluation progresses. If recruitment is slow or practical circumstances change, the sample will be topped up or change to a pragmatic convenience sample, recognising the limitation that those who participate are more likely to have positive experiences with the service.

The interviews will be one-to-one with an experienced qualitative researcher over MS Teams and will last up to ~30 minutes to an hour. They will be semi-structured, where some questions are determined in advance, and some are guided by participants’ answers. The topic guides, which following best practice will be iterated after every few interviews, will be developed by [author one] following her expertise, knowledge of the literature, and some questions will be inspired by Normalisation Process Theory^9^, which is a sociological framework to assess how new interventions become embedded in the real world. The general focus of the topic guides will be on experiences with the service, acceptability, and barriers and/or facilitators to participation and implementation. There will also be reflection on how the service compares to what was offered previously. This will meet outcomes 4 and 10-14 and may corroborate the quantitative findings for outcomes 2, 3, and 5-7.

The data will be audio-recorded and transcribed verbatim by the MS Teams transcription plug-in, which is approved for use at the University of [redacted]. The transcripts will be checked for accuracy and pseudonymised and then the audio-recordings will be deleted. All data will be held on our study one-drive, which only approved team members have access to. Consent from participants will be obtained to hold the transcripts for three years after the end of this study (approximately 2028).

Throughout the qualitative part of the evaluation, we will follow Schwandt, Lincoln, and Guba’s^10^ recommendations for maintaining quality and trustworthiness, which includes keeping a reflexivity log and having regular team discussions.

### Data Analysis

*Quantitative*

We will summarise the quantitative data using descriptive statistics (frequencies, means, medians, measures of variance).

#### Qualitative

We will use descriptive thematic analysis to provide a comprehensive summary of themes and patterns within the transcribed qualitative data without going beyond participants’ words to a higher-order interpretative or theoretical level. This approach is suitable here because there is no understanding of how the service may function in practice. It meets the aim of the interviews to produce a thick description of participants’ experiences and their acceptability towards the service, rather than to interrogate the sociological processes at play. Descriptive thematic analysis also allows us to stay close to participants’ original words, making it ideal for this context where practical, actionable insights are needed to inform the service design within a relatively quick timeframe.

The descriptive thematic analysis will be led by an experienced qualitative researcher who will hold regular ‘coding clinics’ with the research team to share ongoing codes, analytic thinking, and provide opportunity for reflexivity. The analysis will take place on the most up-to-date version of NVivo for Windows and will follow Sandelowski’s^11^ established approach: (1) reading transcripts to be familiar with the content; (2) coding each line or paragraph of data using participants own words; (3) merging codes into categories to reflect patterns of codes; (4) using the one-sheet-of-paper method to manually map important categories to the evaluation aims to identify the thematic narrative and key issues at stake; (5) producing the report.

#### Triangulation

The quantitative and qualitative findings will be integrated to provide a comprehensive understanding of the feasibility, acceptability, other factors influencing the implementation of the service and the impact of the new tobacco dependency services. This will include:

- Triangulation of data to validate findings from multiple sources.
- Development of evidence-based recommendations to enhance service delivery and inform future strategies.
- Dissemination may include meetings with the local council, publication of a peer-reviewed paper in an academic journal, and presentations at academic conferences.

## Background details

### Evaluation team

[redacted]

### Project Partners

[redacted]

### Funding

[Redacted]

### Declarations of interest

None to declare

## References

1. Tobacco and Vapes Bill, House of Commons (2025). <https://bills.parliament.uk/bills/3879>

2.	West R. The very best support for stopping smoking. <http://www.uknscc.org/uknscc2016_presentation_442.php>

3.	McEwen A, Montgomery S, Papadakis S. *Standard Treatment Programme*. 2019. <https://www.ncsct.co.uk/publications/ncsct-standard-treatment-programme>

4.	NICE. *Tobacco: preventing uptake, promoting quitting and treating dependence*. 2023. <https://www.nice.org.uk/guidance/ng209>

5.	Smokers urged to swap cigarettes for vapes in world first scheme. 2023. <https://www.gov.uk/government/news/smokers-urged-to-swap-cigarettes-for-vapes-in-world-first-scheme>

6.	Papadakis S, McEwen A. *Very brief advice on smoking PLUS (VBA+)*. 2021. <https://www.ncsct.co.uk/publications/VBA>+

7.	[redacted].

8.	Malterud K, Siersma VD, Guassora AD. Sample size in qualitative interview studies: guided by information power. Qualitative health research. 2016 Nov;26(13):1753-60.

9.	Murray E, Treweek S, Pope C, MacFarlane A, Ballini L, Dowrick C, Finch T, Kennedy A, Mair F, O'Donnell C, Ong BN. Normalisation process theory: a framework for developing, evaluating and implementing complex interventions. BMC medicine. 2010 Oct;8:1-1.

10.	Schwandt TA, Lincoln YS, Guba EG. Judging interpretations: But is it rigorous? Trustworthiness and authenticity in naturalistic evaluation. New directions for evaluation. 2007 Jun 1;2007(114).

11.	Sandelowski M. Whatever happened to qualitative description?. Research in nursing & health. 2000 Aug;23(4):334-40.
