# Supplementary material for: Implementing a smoking cessation service in routine and manual workplaces: A qualitative service evaluation
Source: Public Health Pract (Oxf). 2026 Oct 3;12:100862. doi: 10.1016/j.puhip.2026.100862 (PMC13678017; doi:10.1016/j.puhip.2026.100862)
Supplement: Supplementary file 2 [file mmc2.docx]

## Supplementary File 2 – Interview Topic Guides

**Manager/Employer Topic Guide**

| **Topic Area** | **Questions** | **Potential Prompts** |
| --- | --- | --- |
| Setting the Scene | Before we start, can you tell me about your workplace? | -Role.  -Number of Staff.  -Tasks people carry out. |
| Smoking in the Workplace Before Introducing the Stop Smoking Support | Thinking back to before the stop smoking support was introduced, how would you describe smoking behaviors in your workplace?      How did these smoking behaviors impact your workplace?    What were the workplace policies around smoking at that time? | - How many employees do you think smoked in your workplace before the service was introduced?  -Where did people tend to smoke?      -Productivity.  -Public Image.    -Impact of these policies.  -How they were perceived.  -Enforcement. |
| Why the Employer Signed Up | What were your reasons for agreeing to offer a stop smoking service within your workplace? (as a company or as an employer)      What were you hoping to achieve by introducing this service into your workplace?    How do you feel about stop smoking support being offered within a workplace setting?    From your point of view, can you recall having any concerns before the service was introduced? | - Was it driven by the company, employee requests, or a combination?  - How does this initiative align with the company's broader approach to employee wellbeing?    -Compared to if employees had to seek it out externally (e.g., from a GP or community service)?    -From your point of view, is there a shared understanding between managers and employees who smoke, of why this stop smoking service is being offered?    -What reassured them? |
| Integration of the Support | Can you tell me what you understand employees are being offered as part of the stop smoking support?            How does the support logistically fit within this workplace?                What is your sense of uptake of the service? | -How do you feel about what is being offered?  -How is the service advertised to employees?  -How does it compare to what was previously offered?      -Where are appointments held?  -How are employees able to access it during work hours?  -Are there any aspects that feel difficult to integrate?  -What have you learned along the way about how to integrate the service? |
| Own Interactions | Have you personally had contact with the stop smoking advisor?    Have there been any changes in workplace policies related to smoking since speaking to the advisor? | -Can you tell me about these interactions?  - Why not?    -Impact. |
| Changes to the Workplace | What feedback have you had from employees who have used this service?    Have you observed or heard about any changes in employees' smoking behaviors since the stop smoking support became available? | - From your point of view, is there a noticeable difference in the number of people who smoke?  - Or to address the previous impact from smoking. |
| Looking Ahead | How valuable do you feel the introduction of the stop smoking service has been?    What are your suggestions for improving the stop smoking service in the future?    How do you see the stop smoking service evolving within this workplace over the next few years? | -Permanent features. |

**Smoking/Tobbaco Cessation Advisor Topic Guide**

| **Topic Area** | **Questions** | **Potential Prompts** |
| --- | --- | --- |
| Setting the Scene | Can you start by telling me about your role as a Tobacco Dependency Advisor? | -What does a typical day look like for you?    -What workplaces have you had contact with? |
| Initial Insights | When you first began working with [Workplace Name], what were your initial impressions of the workplace culture around smoking?    What did you perceive as the main challenges for introducing a stop smoking service into this type of setting?    What is your understanding of why a stop smoking service is being introduced into routine and manual workplaces?    How did you feel about doing this work in a routine and manual setting?    From your point of view, was there anything you did differently knowing it is a routine and manual workplace? | -Logistics.        From your perspective, do you feel you have the right skills and training to help people in the routine and manual workplace setting?      -Mention finances more.  -More support/ less support. |
| Working with Employees | Can you describe your experience of delivering stop smoking support directly to employees within [Workplace Name]?    Can you walk me through a typical consultation?    What has been the general level of employee engagement with the service?    What challenges have you encountered when working with employees?        What aspects of the service have you found to be most effective? | -Format.  -Location.  -Frequency.    -What was offered.      - What types of employees are most likely to seek support?    - Time constraints, shift patterns, stress, peer pressure, privacy, motivation levels.  -How have you adapted your approach to addressing these issues? |
| Working with Employers | What has been your role in collaborating with employers to develop or adapt stop smoking policies?    What have been the challenges when collaborating with employers?    What strategies or approaches have you found most effective in fostering a productive partnership with employers? | - How often do you meet with employers?  - What specific policy areas have you focused on?  -What was employers' attitude towards accessing this type of support?    -How have you attempted to overcome these problems? |
| Impact | What kind of impact do you believe the integrated stop smoking service is having on employees and [Company Name] as a whole? | **-** Have there been any unexpected/negative consequences? |
| Looking Ahead | What are your suggestions for improving or the stop smoking service in these types of workplaces moving forward?    How do you see the stop smoking service evolving within this workplace over the next few years? |  |

**Coordinators Topic Guide**

| **Topic Area** | **Questions** | **Potential Prompts** |
| --- | --- | --- |
| Introduction to Service Provider/Council | Can you start by telling me about the service provider?    What is your role within the service/council? | -Their main mission.  -What work they have done previously in the community? |
| Project Origins | Could you walk me through when the idea of bringing stop smoking support into routine and manual workplaces was first put to you?    What was your understanding of why this support was needed in these workplaces?    What was your role within delivering or implementing the support? | -Who bought the idea to you?  -What were your initial impressions?  -Can you recall having any initial hesitations?    -What do you hope the programme will achieve? |
| The Service | What exactly is the 'offer' to the worksite? Is there a workplace element and an employee element?    How did you decide that this was what was going to be offered to workers?    How does this specific workplace smoking project differ from the 'standard' or 'usual' health interventions the service provider delivers?    What was the plan for where this support was going to be delivered in the workplace?    What was the plan for advertising the support to workers?    Can you recall anything you considered offering that would be specific to routine and manual workplaces?    Can you recall anticipating any challenges with offering this type of support in these settings?    Can you recall there being a discussion of how you would determine that the workplace support was successful? | -Who delivers this support?  -How long is the support going to be delivered in the workplaces?  -Are there different versions of the offer depending on the workplace or worker?  -Behavioural support, pharmacological therapy, counselling, motivational interviewing, quit dates, etc.                -e.g., in break rooms.  -Can you recall there been any deviations from this plan? Why?            -e.g., practicalities, advertising, friction with the manger. |
| The Logistics (Companies) | Tell me about the type of workplaces you would approach?    Could you walk me through your 'pitch' when you first contacted a manager or business owner?    Tell me about your experience visiting sites?    How have you found communication with these sites?    What has been the reaction from companies so far?    What would you say has been the main concern from businesses?    What would you say has been the main appeal?      Have there been any specific requirements or targets from the council that influenced your approach? | -How would you identify these workplaces?  -How would you approach these workplaces?  -What is your definition of routine and manual?                What would you say is the main 'selling point' you use to get them to say yes?    From your point of view, are there any differences between large and small business?  -What would you say has been the main barrier to entry? |
| The Logistics (Service Provider) | Tell me about the logistics on the service provider’s side?    How is it balancing this project with the other projects that the service provider are delivering in the community? | -What are the barriers you experienced?    -What are the facilitators you experienced? |
| Outcomes | Based on your monitoring, what outcomes have you seen from the service so far?    Have you had any feedback from managers or business owners? | -What feedback are you getting from advisors at the worksites?    -Can you give an example of how the service has been 'tweaked' or changed based on feedback from the workers or managers so far? |
| Wind down | To summarise our interview, what would you say has been your main learning point throughout this process? |  |
