# Supplementary material for: Implementing a smoking cessation service in routine and manual workplaces: A qualitative service evaluation
Source: Public Health Pract (Oxf). 2026 Oct 3;12:100862. doi: 10.1016/j.puhip.2026.100862 (PMC13678017; doi:10.1016/j.puhip.2026.100862)
Supplement: Supplementary file 3 [file mmc3.docx]

## Supplementary File 3 – Rapid Analysis Mapped to the Thematic Analysis

| **Thematic Analysis Theme** | **Rapid Analysis Facilitator Theme** | **Rapid Analysis Barrier Theme** | **Illustrative Quote** |
| --- | --- | --- | --- |
| Coherence:  Misunderstandings of the Service | Appeal of the service being free. | Assumption from some workplaces that service implementation would introduce extra work.  Confusion between intended and actual service scope. | “W*hen people are approaching these businesses, they're asking for different things, and some might for donations. Where we're like**, actually, no, we don't want to take anything from you. We want to give you stuff, we're not taking; we're giving you guys an extra tool*.” *(Service Coordinator)* |
| Cognitive Participation:  Navigating Organisational Gatekeeping | Invitation from a perceived authoritative source.  Highlighting the potential positive impact on the business. Keeping email exchanges short.  Persistence when communicating with potential workplaces. | Identifying and engaging the right manager at the right time to agree to service implementation. Limited time for relationship building prevented site recruitment. | “*It's more complicated for a place like* [supermarket]*, for example. It's more complicated than for a smaller business or a smaller shop where you can just go in and talk to the owner of the shop*.” *(Council Coordinator)* |
| Collective Action:  Managing Workplace Logistics | Advertising extensively through multiple channels. | Limited workspace for on-site clinics and confidentiality concerns reduced employee engagement.  Availability of appointments was incongruent with some employees' working hours. | *“We can have a space next to a checkout station. I think in the* [customer-facing organisation] *that we work with, we're stationed quite close to everything. It's quite open. It's not always as confidential, which can be a drawback.” (Service Coordinator)* |
| Reflexive Monitoring:  Long-Term Sustainability of the Service | Lack of communication and contingency between managers at the workplaces.  Lack of communication and understanding of service impact on cessation outcomes. | Delivering smoking cessation support to employees is good in theory. | “*I’d love to be able to say with confidence that X number of staff have attended a smoking cessation clinic and 80% of those have now quit smoking.” (Manager)* |
